# Genome-guided insight into the methylotrophy of *Paracoccus aminophilus* JCM 7686

**DOI:** 10.3389/fmicb.2015.00852

**Published:** 2015-08-21

**Authors:** Lukasz Dziewit, Jakub Czarnecki, Emilia Prochwicz, Daniel Wibberg, Andreas Schlüter, Alfred Pühler, Dariusz Bartosik

**Affiliations:** ^1^Department of Bacterial Genetics, Institute of Microbiology, Faculty of Biology, University of WarsawWarsaw, Poland; ^2^Institute for Genome Research and Systems Biology, Center for Biotechnology (CeBiTec), Bielefeld UniversityBielefeld, Germany

**Keywords:** *Paracoccus aminophilus* JCM 7686, methylotrophy, serine cycle, methanol, methylated amine, *N,N*-dimethylformamide, chromid, plasmid

## Abstract

*Paracoccus aminophilus* JCM 7686 (*Alphaproteobacteria*) is a facultative, heterotrophic methylotroph capable of utilizing a wide range of C1 compounds as sole carbon and energy sources. Analysis of the JCM 7686 genome revealed the presence of genes involved in the oxidation of methanol, methylamine, dimethylamine, trimethylamine, *N*,*N*-dimethylformamide, and formamide, as well as the serine cycle, which appears to be the only C1 assimilatory pathway in this strain. Many of these genes are located in different extrachromosomal replicons and are not present in the genomes of most members of the genus *Paracoccus*, which strongly suggests that they have been horizontally acquired. When compared with *Paracoccus denitrificans* Pd1222 (type strain of the genus *Paracoccus*), *P. aminophilus* JCM 7686 has many additional methylotrophic capabilities (oxidation of dimethylamine, trimethylamine, *N,N*-dimethylformamide, the serine cycle), which are determined by the presence of three separate gene clusters. Interestingly, related clusters form compact methylotrophy islands within the genomes of *Paracoccus* sp. N5 and many marine bacteria of the *Roseobacter* clade.

## Introduction

The genus *Paracoccus* (*Alphaproteobacteria*) comprises bacteria isolated from various pristine and polluted environments (e.g., soil, marine sediments, seawater, biofilters, activated sludge, or human tissues) (Urakami et al., 1990; Siller et al., 1996; Lipski et al., 1998; Tsubokura et al., 1999; Funke et al., 2004; Lee et al., 2004; Liu et al., 2006). These bacteria show diverse metabolic properties and can switch between different growth modes—for example, heterotrophic growth on a wide range of organic compounds vs. chemolithoautotrophic growth on reduced sulfur compounds, hydrogen or ferrous ions as the energy sources, or aerobic respiration vs. anaerobic nitrate respiration (Kelly et al., 2006). Because of their degradative capabilities, many *Paracoccus* spp. strains are suitable for application in bioremediation systems. They have been successfully employed in the bioremediation of soils contaminated with polycyclic aromatic hydrocarbons (PAHs, Sun et al., 2013) and in the removal of nitrate and *N,N*-dimethylformamide (DMF) from wastewater (Liu et al., 2012; Sanjeevkumar et al., 2013). Moreover, their ability to utilize pesticides and insecticides, including the highly toxic chlorpyrifos, 3,5,6-trichloro-2-pyridinol, methyl parathion, and carbonfuran, has also been demonstrated (Li et al., 2011).

About 50% of known *Paracoccus* spp. strains are described as methylotrophs, i.e., organisms utilizing C1 compounds (reduced carbon compounds containing no carbon-carbon bonds) as sole carbon and energy sources (Baker et al., 1998). Methylotrophs play an important role in global carbon, nitrogen, and sulfur cycling, and for this reason their biochemistry has been subjected to extensive studies (Trotsenko and Murrell, 2008; Chistoserdova, 2011). As described by Chistoserdova (2011), the methylotrophy process can be divided into three stages: (i) primary oxidation of C1 substrates, which results in formaldehyde, methyl- or methylene-tetrahydrofolate (CH_3_–THF or CH_2_ = THF) formation, (ii) oxidation of formaldehyde, CH_3_–THF or CH_2_ = THF to CO_2_, and (iii) assimilation of C1 units. The final stage may be performed via the ribulose monophosphate (RuMP) cycle, the serine cycle or the Calvin–Benson–Bassham (CBB) cycle (Chistoserdova, 2011).

*Paracoccus denitrificans* Pd1222 (type strain of the genus *Paracoccus*, Kelly et al., 2006) exemplifies so-called autotrophic methylotrophs (Chistoserdova, 2011), assimilating CO_2_ derived from the oxidation of C1 compounds [in Pd1222 these are methanol (MeOH) or methylamine (MA)] via the Calvin cycle (Baker et al., 1998). Other methylotrophic strains of *Paracoccus* spp. have been poorly characterized. Interestingly, in comparison with *P. denitrificans*, these strains show significant differences in their methylotrophic metabolism, not only in the range of C1 compounds utilized [many strains are able to oxidize trimethylamine (TMA), trimethylamine *N*-oxide (TMAO), dimethylamine (DMA), dichloromethane or DMF] (Urakami et al., 1990; Kim et al., 2001, 2003; Turova et al., 2001), but also in the central metabolic pathways mediating C1 unit assimilation (Beck et al., 2015).

Very recently, a set of genes encoding the enzymes of the serine cycle have been identified in *Paracoccus* sp. N5 (Beck et al., 2015). Since this strain also encodes all enzymes of the Calvin cycle it was classified as a facultatively autotrophic methylotroph (Beck et al., 2015). In this work we have characterized *Paracoccus aminophilus* JCM 7686, which represents another metabolic group of methylotrophic bacteria—the heterotrophic serine cycle methylotrophs. This strain does not encode ribulose-1,5-bisphosphate carboxylase/oxygenase (RuBisCO), crucial for the Calvin cycle, so its methylotrophic properties exclusively rely on the serine cycle.

The multipartite genome of *P. aminophilus* JCM 7686 was described in detail in our previous report (Dziewit et al., 2014). It is composed of a single circular chromosome (3.6 Mb) and eight circular extrachromosomal replicons. The functional analyses of those replicons revealed that six of them (pAMI1, pAMI2, pAMI3, pAMI4, pAMI7, and pAMI8) are plasmids and two (pAMI5 and pAMI6) are chromids (i.e., elements essential for host viability and sharing characteristics of both chromosomes and plasmids) (Dziewit et al., 2007, 2011a,b, 2014). In the present study, bioinformatic sequence analyses of the JCM 7686 genome together with functional characterization of selected genes have provided deeper insight into both the biochemistry of the methylotrophy of this strain and the role of the extrachromosomal genetic elements in determination of its methylotrophic capability.

## Materials and methods

### Strains, plasmids, and culture conditions

The strains used in this study are described in Table S1. All strains were grown in lysogeny broth (LB) (Sambrook and Russell, 2001) at 37°C [*E. coli* TG1 (Gibson, 1984) and S17-1 (Simon et al., 1983)] and 30°C [*P. aminophilus* JCM 7686R (Bartosik et al., 2002)]. *P. aminophilus* was also grown in minimal salts medium (AC) (Wood and Kelly, 1977) at 30°C. When necessary, the media were supplemented with kanamycin (50 μg/ml), tetracycline (2 μg/ml for *Paracoccus* spp. or 20 μg/ml for *E. coli*), chloramphenicol (12.5 μg/ml), rifampicin (50 μg/ml), gentamicin (10 μg/ml), or sucrose (11% w/v). The following compounds were used as the carbon source in minimal media: L-arabinose (0.2% w/v), *N,N*-dimethylformamide (20 mM), trimethylamine (20 mM), dimethylamine (10 mM), methylamine (10 mM), formamide (20 mM), and methanol (20 mM). All plasmids used [i.e., pBBR1MCS-3 (Kovach et al., 1994), pBBR1MCS-5 (Kovach et al., 1994), pDIY-KM (Dziewit et al., 2011a), pDS132 (Philippe et al., 2004), pKRP12 (Reece and Phillips, 1995), pRK2013 (Ditta et al., 1980)] and constructed in this study are described in Table S2.

### Standard genetic manipulations

The isolation of DNA and common DNA manipulation methods were performed as described by Sambrook and Russell (2001). PCR was performed in an Eppendorf Mastercycler with pDIY-KM, pKRP12 or total DNA of *P. aminophilus* as the template, appropriate oligonucleotide primers (Table S3), dNTP mixture and Phusion High-Fidelity DNA polymerase (Thermo Scientific; with supplied HF buffer). Transformation of *E. coli* strains was performed according to the method of Kushner (1978). Bi- and tri-parental matings were performed as previously described (Bartosik et al., 2001). For triparental mating, overnight cultures of the donor strain *E. coli* TG1, the appropriate recipient strain, and *E. coli* DH5α carrying the helper plasmid pRK2013 were mixed in a ratio 1:2:1. For biparental mating, overnight cultures of the donor strain *E. coli* S17-1 carrying a mobilizable vector and the appropriate recipient strain were mixed in a ratio 1:1. Then 100 μl of the mixture was spread on a plate with solidified LB medium and incubated overnight at 30°C. Then the bacteria were washed off the plate, and the suitable dilutions were plated on appropriate selective media to select transconjugants (Bartosik et al., 2001).

### Gene disruption

Deletion of selected *P. aminophilus* JCM 7686 genes was performed using a gene replacement method. DNA cassettes for gene disruption, containing an antibiotic resistance gene flanked by PCR-amplified DNA fragments (ca. 500 bp) homologous to the DNA regions surrounding the gene targeted for disruption, were created by overlap extension PCR with specific primers (Table S3) or by restriction cloning (Table S2). The obtained DNA cassettes were transferred by biparental mating from *E. coli* S17-1 into rifampicin resistant *P. aminophilus* JCM 7686R cells on *sacB* gene-containing vector pDS132, unable to replicate in *Alphaproteobacteria*. Double-cross recombinants were selected on appropriate medium containing rifampicin, sucrose and kanamycin or tetracycline, depending on the antibiotic resistance gene used for cassette construction. The correctness of the generated disruptions was verified by sequencing DNA fragments amplified by PCR using appropriate primer pairs (Table S3).

### RT-qPCR

Total RNAs used for the RT-qPCR analyses were isolated from *P. aminophilus* cells grown on TMA or arabinose as the sole carbon and energy source. The strain was cultured in appropriate minimal media in three biological repeats and cells were collected during late exponential phase. RNA was isolated using TRI Reagent® Solution (Ambion) according to the manufacturer's recommendations. Contaminating DNA was removed using DNA-free™, DNase Treatment & Removal (Ambion) according to the manufacturer's recommendations. The total RNA was transcribed into cDNA using the Maxima First Strand cDNA Synthesis Kit for RT-qPCR (Thermo Scientific) according to the manufacturer's instruction. RT-qPCR reactions were carried out in LightCycler® 480 Instrument II (Roche) using 5 × HOT FIREPol® EvaGreen® qPCR Mix Plus (no ROX). Oligonucleotide primers used in the study are listed in Table S3. Relative quantification of gene transcription was performed using the comparative *C*_*t*_ (threshold cycle) method.

### Bioinformatics

The putative function of particular genes was assigned using BLAST programs (Altschul et al., 1997) and PRIAM tool (Claudel-Renard et al., 2003) as previously described (Heinl et al., 2012). Metabolic pathways were recognized and described using Pathway tools (Karp et al., 2010), the MetaCyc database (Caspi et al., 2008) and GenDB 2.0 (Meyer et al., 2003). For the identification of methylotrophy-linked genes/proteins a BLASTn/BLASTp analysis comparing the sequence of each gene/protein with the genome/proteome of a particular *Paracoccus* strain was performed. Strict cutoff values were applied for this analysis: *e* < 10^−40^, 70% for minimal query coverage and sequence identity of at least 45%.

## Results and discussion

### Identification of *P. aminophilus* JCM 7686 genes linked to methylotrophy

**Figure 1:**
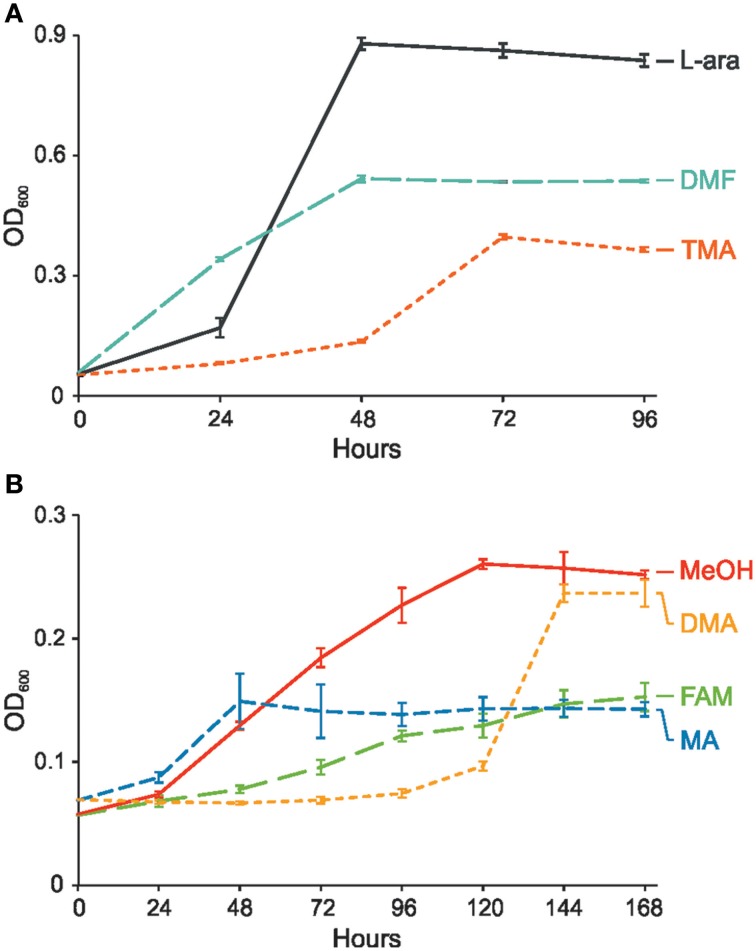
**Growth of ***P. aminophilus*** JCM 7686 on various C1 compounds or L-arabinose as a sole carbon and energy source. (A)** Growth on L-arabinose (L-ara), *N,N*-dimethylformamide (DMF), and trimethylamine (TMA), **(B)** Growth on methanol (MeOH), dimethylamine (DMA), formamide (FAM), and methylamine (MA). The values are means of three replicates, and the error bars indicate the standard deviations.

**Figure 2:**
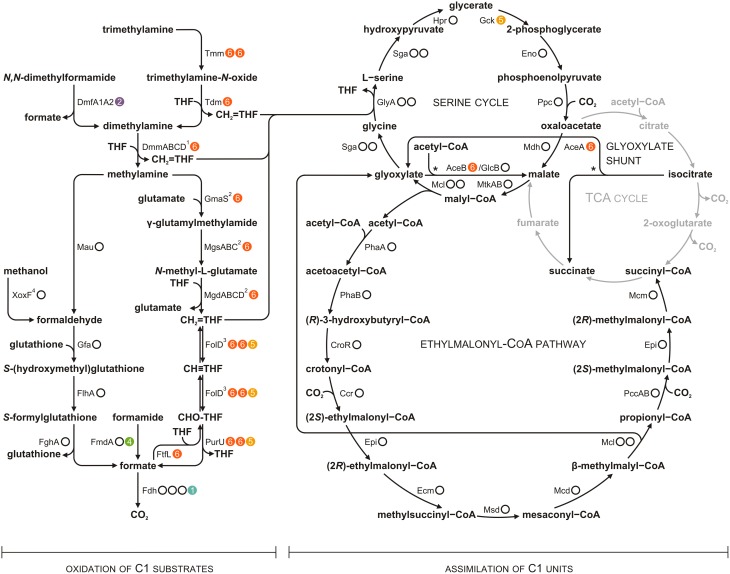
**Methylotrophic metabolism of ***P. aminophilus*** JCM 7686 reconstructed on the basis of genomic analysis**. Dots to the right of the enzyme designations indicate the number of loci encoding the corresponding protein and their location in the genome (white dots, the chromosome; colored dots with numbers, extrachromosomal replicons pAMI1, pAMI2, pAMI4, pAMI5 or pAMI6). Full names of enzymes are given in Table S4. The glyoxylate shunt reactions are indicated by asterisks. ^1^DmmABCD is proposed to be involved in DMA metabolism on the basis of mutational analysis (Beck et al., 2015 and this study). ^2^The presented *N*-methylglutamate pathway (NMGP) was selected from among the possible schemes. Not only CH_2_ = THF, but also free formaldehyde is considered to be a product of this pathway (Nayak and Marx, 2014). ^3^Previously, FolD was thought to act only in the oxidative direction (Chistoserdova, 2011), but it has recently been proposed to act bidirectionally, at least in some bacteria (Beck et al., 2015). ^4^XoxF-type dehydrogenases may convert methanol directly into formate (Keltjens et al., 2014).

*P. aminophilus* JCM 7686 was isolated in Japan from soil contaminated with *N,N*-dimethylformamide (DMF) as a strain able to utilize many C1 compounds (Figure S1) (Urakami et al., 1990). However, no further analysis regarding the methylotrophy of this strain was performed. In the initial stage of this study we confirmed that, besides DMF, JCM 7686 can utilize methylamine, dimethylamine, trimethylamine, and formamide. We also found that it is able to utilize methanol (Figure 1), which is contrary to the original observations of Urakami et al. (1990). To determine the genetic basis of these phenotypes we examined the JCM 7686 genome (Dziewit et al., 2014) for the presence of genes linked to C1 metabolism and we performed functional analysis of selected genes to confirm our predictions. The collected data permitted reconstruction of the complex C1 metabolic pathway of this strain (Figure 2, Table S4).

### Genes involved in methanol utilization

The ability of *P. denitrificans* Pd1222 to utilize methanol was shown to be dependent on the presence of *mxa* genes encoding i.a. subunits of a heterotetrameric PQQ-dependent calcium-binding MeOH dehydrogenase (MxaFI) (Van Spanning et al., 1991). Although *P. aminophilus* JCM 7686 is also able to oxidize MeOH, the *mxa* genes were not detected in this strain. Nevertheless, a gene encoding another type of MeOH dehydrogenase (*xoxF*; JCM7686_0090) was identified within the JCM 7686 chromosome. XoxF represents a group of homodimeric methanol dehydrogenases, related to the large subunit of MxaFI, which bind rare-earth elements instead of calcium (Keltjens et al., 2014). Interestingly, *xoxF* genes are widespread among both methylotrophs and non-methylotrophic bacteria (Keltjens et al., 2014). Studies on *Rhodobacter sphaeroides* revealed that XoxF is required for methanol oxidation during both aerobic and anaerobic photosynthetic growth (Wilson et al., 2008).

The *P. aminophilus*-encoded XoxF belongs to the XoxF5 protein family (Keltjens et al., 2014). In the JCM 7686 chromosome, the *xoxF* gene is clustered together with *xoxG* (JCM7686_0091, encoding a cytochrome c used as an electron acceptor for methanol oxidation) and *xoxJ* (JCM7686_0092, encoding a putative periplasmic binding protein), as well as with the genes of a glutathione-dependent formaldehyde dehydrogenase system (JCM7686_0085, JCM7686_0086, and JCM7686_0089) (Table S4). It suggests that formaldehyde, and not formate, is the product of methanol oxidation catalyzed by XoxF5 proteins (Keltjens et al., 2014). The clustering of *xoxF* and the genes of the glutathione-dependent formaldehyde oxidation pathway is also seen in many other alphaproteobacterial genomes, e.g., in *P. denitrificans* Pd1222, *Roseobacter litoralis* Och 149, *Dinoroseobacter shibae* DFL 12 and *Sinorhizobium fredii* HH103 (Keltjens et al., 2014 and this study).

**Figure 3:**
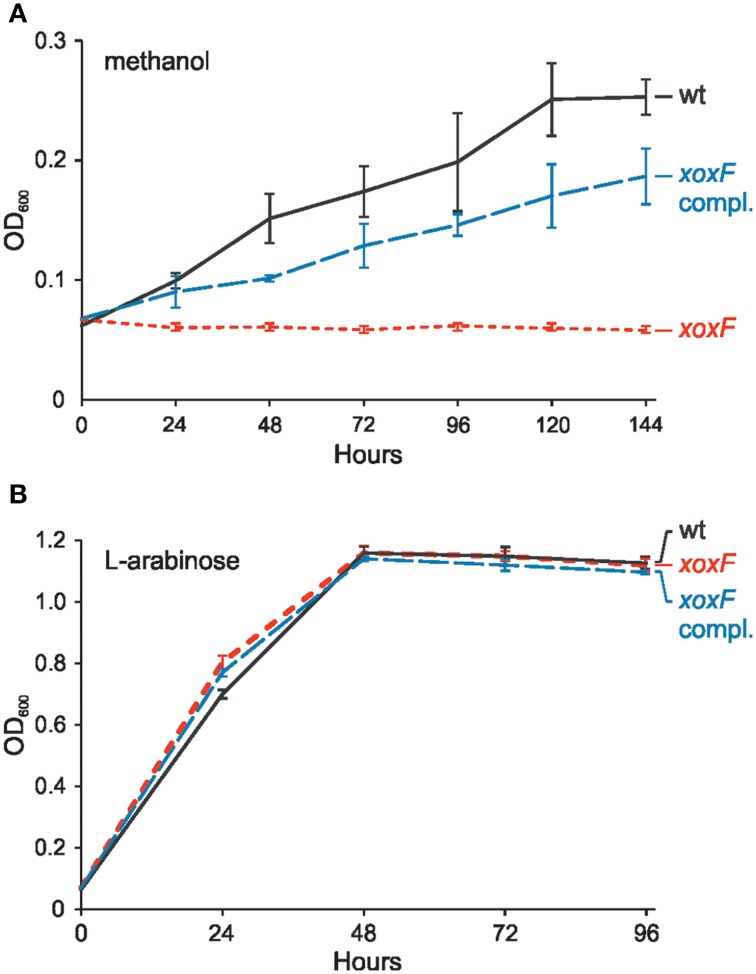
**Effect of the ***xoxF*** mutation on growth of ***P. aminophilus*** JCM 7686 on methanol (A) and L-arabinose (B)**. wt, wild type; *xoxF, xoxF* insertional mutant; *xoxF* compl., *xoxF* insertional mutant complemented with a copy of the *xoxF* gene cloned in pBBR1MCS-3. The values are means of three replicates, and the error bars indicate the standard deviations.

To evaluate the role of the identified *xoxF* gene in methanol utilization, its mutational analysis was performed. This revealed that a strain with disrupted *xoxF* was unable to grow in minimal medium with methanol as the sole source of carbon. The wild-type phenotype was restored when the *xoxF* gene cloned in vector pBBR1MCS-3 was introduced into the mutant cells (Figure 3). This confirmed that XoxF is the enzyme responsible for methanol oxidation in *P. aminophilus* JCM 7686.

### Genes involved in utilization of methylated amines

Three gene clusters, potentially involved in the utilization of TMA, DMA and MA as carbon, nitrogen and energy sources were identified in the *P. aminophilus* genome. Two of them are located within the chromid pAMI6 and contain genes encoding enzymes responsible for TMA oxidation via trimethylamine *N*-oxide (TMAO) and MA oxidation via *N*-methylglutamate. The third cluster is located within the chromosome and encodes methylamine dehydrogenase.

**Figure 4:**
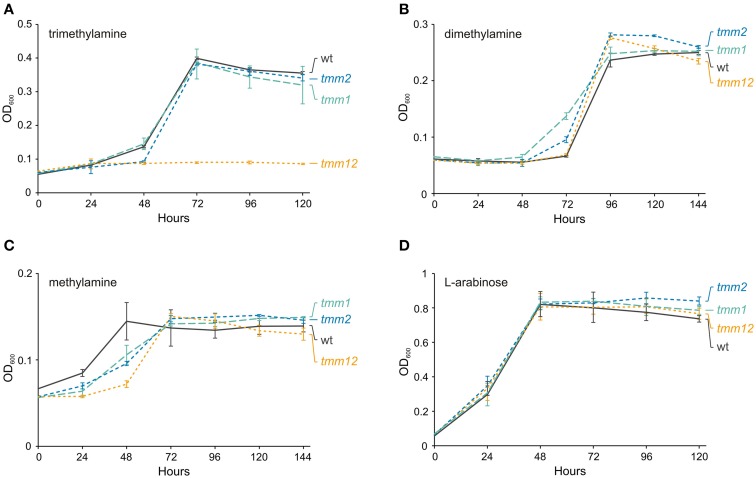
**Effect of the ***tmm1*** and ***tmm2*** mutations on growth of ***P***. ***aminophilus*** JCM 7686 on trimethylamine (A), dimethylamine (B), methylamine (C), and L-arabinose (D)**. wt, wild type; *tmm1, tmm1* insertional mutant; *tmm2, tmm2* insertional mutant; *tmm12*, double insertional mutant in *tmm1* and *tmm2* genes. The values are means of three replicates, and the error bars indicate the standard deviations.

TMA oxidation via TMAO depends on the activity of three enzymes: (i) TMA monooxygenase, (ii) TMAO demethylase, and (iii) DMA monooxygenase. Interestingly, pAMI6 carries two genes, *tmm1* (JCM7686_pAMI6p076) and *tmm2* (JCM7686_pAMI6p102), encoding putative TMA monooxygenases. The predicted Tmm1 and Tmm2 proteins show high amino acid (aa) sequence similarity (75%) to one another and share about 60% identity with the Tmm protein of *Methylocella silvestris* BL2 (Chen et al., 2011). To analyze the role of the *tmm* genes in the TMA metabolism of JCM 7686, three mutant strains were constructed lacking either *tmm1* or *tmm2*, or both genes. The growth rate of the strains containing single mutations (*tmm1* or *tmm2*) in minimal medium with TMA as the sole carbon and energy source was identical to that of the wild type strain (Figure 4). In contrast, the double mutant strain (*tmm1tmm2*) was no longer able to utilize TMA, but it still had the ability to grow on medium supplemented with DMA or MA (the products of TMA utilization) (Figure 4). The growth on TMA was restored when *tmm1* or *tmm2* gene cloned in vector pBBR1MCS-5 was introduced into the double mutant strain (data not shown). These results indicated that both identified *tmm* genes encode enzymes with the same specificity and that they are both involved in the first stage of TMA metabolism. Additionally, the contribution of two *tmm* genes in TMA oxidation was confirmed by RT-qPCR. It was shown that transcript levels of both genes are increased in a similar degree during growth on TMA in comparison with non-methylotrophic conditions (Table 1).

**Table 1:** **Changes in the transcript levels of selected methylotrophy genes of ***P. aminophilus*** JCM 7686 in methylotrophic vs. non-methylotrophic conditions determined by RT-qPCR analysis**.

**ORF name**	**Protein name**	**Process**	Fold change ± SD^*^
*tmm1*	TMA monooxygenase	oxidation of TMA	26.7±2.8
*tmm2*	TMA monooxygenase	oxidation of TMA	27.3±1.9
*hpr*	hydroxypyruvate reductase	serine cycle	3.0±1.0
*gck*	glycerate 2-kinase	serine cycle	5.9±0.7
*aceA*	isocitrate lyase	glyoxylate shunt	13.5±2.6
*ecm*	ethylmalonyl-CoA mutase	ethylmalonyl-CoA pathway	4.9±0.6
*mcm*	methylmalonyl-CoA mutase	ethylmalonyl-CoA pathway	4.6±1.0

* *SD, standard deviation*.

Genes encoding TMAO demethylase (*tdm*, JCM7686_pAMI6p069) and DMA monooxygenase (*dmmDABC*, JCM7686_pAMI6p074-71) were identified in the vicinity of *tmm1*. The predicted Tdm of *P. aminophilus* shares 63% aa sequence identity with the Tdm protein of *Ruegeria pomeroyi* DSS-3 (Lidbury et al., 2014) and the DmmDABC proteins are homologous to four subunits of the putative DMA monooxygenase of *Methylocella silvestris* BL2 (Zhu et al., 2014). The DmmC proteins of strains JCM 7686 and BL2 share 66% aa sequence identity, while the three other putative DMA subunits encoded by these strains (DmmA, DmmB, and DmmD) are less well-conserved (39, 41, and 37% aa sequence identity, respectively).

**Figure 5:**
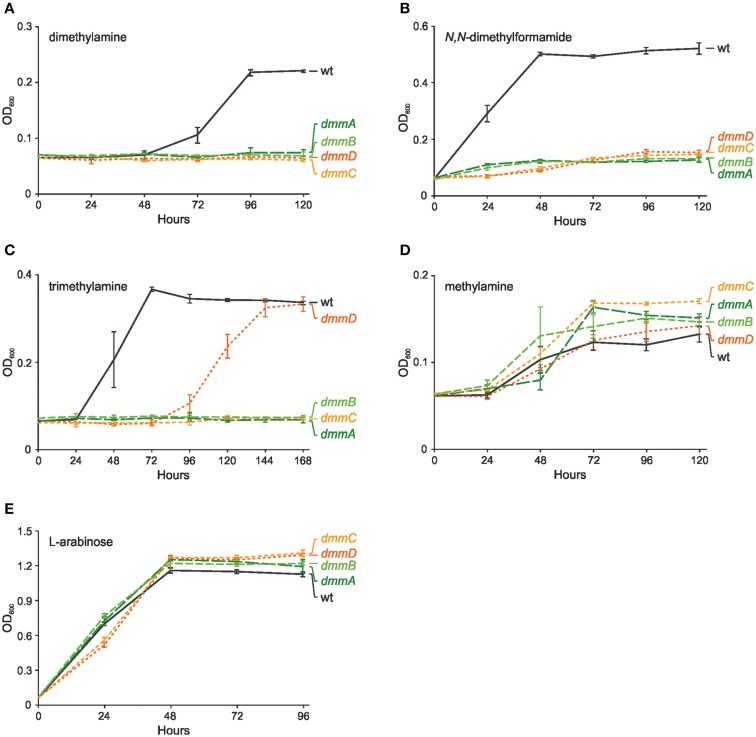
**Effect of mutations in genes encoding four putative subunits of dimethylamine monooxygenase (***dmmDABC***) on growth of ***P. aminophilus*** JCM 7686 on dimethylamine (A), ***N,N***-dimethylformamide (B), trimethylamine (C), methylamine (D), and L-arabinose (E)**. wt, wild type; *dmmA, dmmA* insertional mutant; *dmmB, dmmB* insertional mutant; *dmmC, dmmC* insertional mutant; *dmmD, dmmD* insertional mutant. The values are means of three replicates, and the error bars indicate the standard deviations.

To verify the function of the *P. aminophilus dmmDABC* genes, their mutational analysis was performed. The four mutant strains carrying deletions of the individual *dmm* genes failed to grow on dimethylamine as the sole carbon and energy source, while they showed the same growth rate as the wild-type strain when cultivated on methylamine or L-arabinose (Figure 5). The wild-type phenotype was restored when the *dmmDABC* module cloned in vector pBBR1MCS-3 was introduced into the mutant strains (data not shown). The mutations also influenced growth on C1 compounds that are metabolized via DMA, i.e., *N,N*-dimethylformamide and trimethylamine (Figure 5). Interestingly, inactivation of *dmmD* had a much weaker effect on growth on TMA than the inactivation of the other *dmm* genes (Figure 5). This observation is consistent with the hypothesis that the DmmD protein is not necessary for the conversion of DMA into MA and formaldehyde, but is an auxiliary subunit of the DMA monooxygenase which may convert formaldehyde into methylene-THF (Zhu et al., 2014).

Methylamine (MA), which is the product of DMA utilization, is presumably oxidized by *P. aminophilus* via two different pathways. Upstream and downstream of *tmm2*, we identified genes responsible for MA oxidation via *N*-methylglutamate (the *N*-methylglutamate pathway for MA oxidation, NMGP). They are arranged in two putative operons encoding three enzymes of the pathway: (i) the *mgsABC-gmaS* operon (JCM7686_pAMI6p108-105) encoding glutamate-MA ligase (GmaS) and *N*-methyl-L-glutamate synthase (MgsABC), and (ii) the *mgdABCD* operon (JCM7686_pAMI6p099-096) encoding *N*-methyl-L-glutamate dehydrogenase (MgdABCD). Most of the NMGP enzymes subunits show high levels of aa sequence identity with the respective proteins of *M. silvestris* BL2 (Chen et al., 2010).

The second *P. aminophilus* pathway responsible for MA oxidation relies on the activity of a two-subunit MA dehydrogenase (Mau). Genes encoding this enzyme are arranged in a large chromosomal cluster (JCM7686_0162-0171, *mauFBEDACJGMN*). This gene cluster is very similar to the *mau* region located in plasmid 1 of *P. denitrificans* Pd1222 (approximately 75% nucleotide sequence identity) (van Der Palen et al., 1995). The *mau* genes are crucial for growth of *P. denitrificans* Pd1222 on MA (van Der Palen et al., 1995). However, in *P. aminophilus* we found that mutation of the gene encoding the catalytic subunit MauA did not abolish growth on this compound. In this case, the growth rate on MA-containing medium was reduced (Figure S2), which indicated that both identified pathways for MA utilization are active in *P. aminophilus*.

### Genes involved in utilization of formamides

Strain JCM 7686 can also utilize formamides, including *N,N*-dimethylformamide (Figures 1, 2). Formamide breakdown is most probably catalyzed by two chromosomally- and pAMI4-encoded acetamidases/formamidases (JCM7686_1450, JCM7686_pAMI4p036), while DMF utilization is dependent on a pAMI2-encoded *N,N*-dimethylformamidase (DMFase) (JCM7686_pAMI2p015-017). The latter assumption was confirmed in our previous study in which we found that *P. aminophilus* strain deprived of pAMI2 was unable to utilize DMF (Dziewit et al., 2010). Plasmid pAMI2 carries genes encoding two subunits of DMFase (DmfA1 and DmfA2) which are organized in an operon. More detailed studies revealed that the expression of these genes is activated in the presence of DMF by the LuxR-family transcriptional activator DmfR (Dziewit et al., 2010). Interestingly, a related *dmfA1*-*dmfA2* locus (encoding predicted proteins sharing 34% and 40% aa sequence identity with DmfA1 and DmfA2 of pAMI2, respectively) was also identified within chromid pAMI5 (JCM7686_pAMI5p063-064). However, these genes have not been found to be linked to DMF utilization.

### Genes involved in oxidation of formaldehyde and CH_2_**=**THF to CO_2_ and reduction of formate to CH_2_**=**THF

Most of the aforementioned pathways of C1 substrate oxidation result in the formation of formaldehyde and/or CH_2_ = THF (Chistoserdova, 2011; Keltjens et al., 2014; Lidbury et al., 2014; Nayak and Marx, 2014) (Figure 2). In *P. aminophilus* JCM 7686, the oxidation of formaldehyde to formate is most likely achieved via the glutathione-dependent pathway utilizing three chromosomally encoded enzymes: (i) *S*-(hydroxymethyl)glutathione synthase (Gfa, JCM7686_0085), (ii) *S*-(hydroxymethyl)glutathione dehydrogenase (FlhA, JCM7686_0086), and (iii) *S*-formylglutathione hydrolase (FghA, JCM7686_0089). Each of these enzymes shares a high level of aa sequence identity (~85%) with the corresponding protein from *P. denitrificans* Pd1222. It is worth noting that the *S*-(hydroxymethyl)glutathione dehydrogenase was previously recognized as essential for methylotrophic growth of *P. denitrificans* (Ras et al., 1995).

*P. aminophilus* JCM 7686 encodes enzymes involved in transitions between CH_2_ = THF and formate in both the oxidizing and reducing directions. The oxidation of the methylene group of CH_2_ = THF is associated with energy release, while the reductive pathway is required to supply CH_2_ = THF to the serine cycle (see below) during growth of *P. aminophilus* on methanol and formamide (i.e., C1 compounds, whose oxidation leads to the formation of formaldehyde or formate but not CH_2_ = THF) (Figure 2).

Oxidation of the methylene group to formate is performed by the action of two enzymes: CH_2_ = THF dehydrogenase/CH_2_ = THF cyclohydrolase (FolD) and formyltetrahydrofolate deformylase (PurU). FolD and PurU are encoded by three homologous two-gene loci (*folD-purU*) located in pAMI6 (2 loci) and pAMI5 (Table S4). In the reduction pathway, formate is loaded into THF by formate-tetrahydrofolate ligase (FtfL, JCM7686_pAMI6p042) and then reduced to the methylene group. In many methylotrophs (e.g., *Methylobacterium extorquens*) the reduction process is performed by the sequential action of two enzymes: CH_2_ = THF cyclohydrolase (Fch) and CH_2_ = THF dehydrogenase (MtdA) (Chistoserdova, 2011). Since *P. aminophilus* encodes neither Fch nor MtdA, the reduction of 10-formyl-THF to CH_2_ = THF is presumably catalyzed by FolD which seems to work bi-directionally in some bacteria (Beck et al., 2015).

The product of oxidation of formaldehyde and the methylene group of CH_2_ = THF is formate. It is further oxidized to CO_2_ by formate dehydrogenase (Fdh) (Chistoserdova et al., 2004). In the *P. aminophilus* genome there are four gene clusters encoding formate dehydrogenases; three of them are located in the chromosome (JCM7686_0639-0643, JCM7686_2088, JCM7686_3476-3480) and one in plasmid pAMI1 (JCM7686_pAMI1p027).

### Genes involved in assimilation of C1 units

In contrast to other methylotrophic strains of the genus *Paracoccus, P. aminophilus* JCM 7686 is unable to grow autotrophically using the Calvin cycle. The genome of this strain does not encode subunits of the key enzyme of this process, ribulose-1,5-bisphosphate carboxylase/oxygenase (RuBisCO). Therefore, the only possible way to assimilate C1 compounds seems to be via the serine cycle. *P. aminophilus* carries a chromosomally encoded cluster of serine cycle genes, which is also highly conserved in the genome of *Paracoccus* sp. N5 (Beck et al., 2015). Since many of these genes encode proteins that are highly divergent from the well-studied serine cycle enzymes of other methylotrophs (Beck et al., 2015), functional analysis was required to confirm their specific activities.

The serine cycle gene cluster of *P. aminophilus* is incomplete since it does not contain a *gck* gene encoding glycerate 2-kinase, which is, surprisingly, present at a different genomic location—within chromid pAMI5. Interestingly, the Gck of *P. aminophilus* is more closely related to a protein from the marine bacterium *Labrenzia alexandrii* DFL-11 (72% aa sequence identity) than to the Gck of *Paracoccus* sp. N5 (38% aa sequence identity) and other homologous genes found in *Paracoccus* spp., which strongly suggests that the JCM 7686 *gck* gene was independently acquired by horizontal gene transfer.

The serine cycle cannot operate without regeneration of glyoxylate from acetyl-CoA, which can proceed via the glyoxylate shunt or the ethylmalonyl-CoA pathway (EMCP) (Chistoserdova, 2011). Both pathways also enable growth on C2 compounds. The chromosome of *P. aminophilus* contains the genetic information required for the synthesis of all enzymes of the EMCP (Table S4). Moreover, pAMI6 contains a two-gene locus (JCM7686_pAMI6p120-121) encoding putative enzymes of the glyoxylate shunt: isocitrate lyase (AceA) and malate synthase (AceB). An additional copy of the gene encoding malate synthase (malate synthase G, GlcB, JCM7686_1627) was identified within the chromosome of this strain. Therefore, our bioinformatic sequence analysis indicates that *P. aminophilus* may regenerate glyoxylate via both the EMCP and glyoxylate shunt.

We performed RT-qPCR analyses to confirm that the predicted serine cycle genes (*hpr* carried within pAMI6 and *gck* carried within pAMI5), the gene of isocitrate lyase (*aceA*) involved in the glyoxylate shunt, as well as two genes of the ethylmalonyl-CoA pathway (*ecm* and *mcm*) are linked to methylotrophic metabolism of the JCM 7686 strain. The results showed that in each case the transcript level was elevated during methylotrophic growth (Table 1).

### Abundance of methylotrophy-linked genes in *Paracoccus* spp. genomes

**Figure 6:**
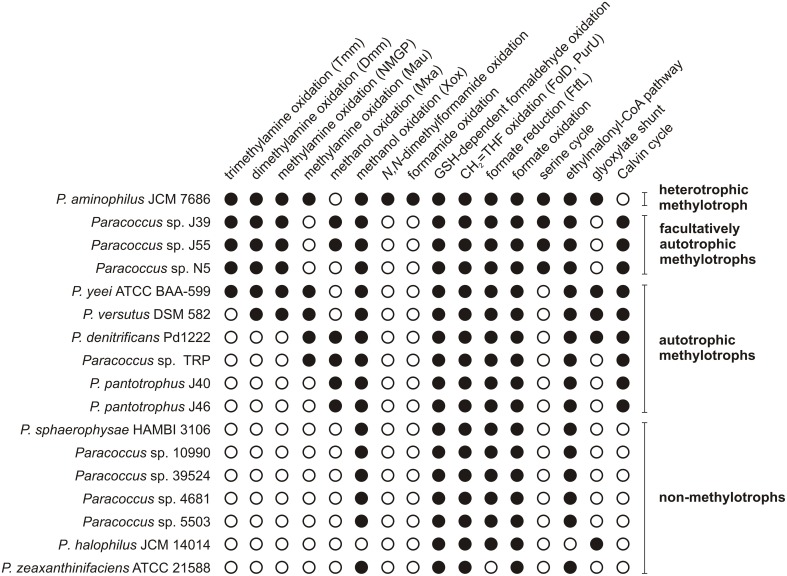
**The methylotrophy-linked metabolic capabilities encoded by the genomes of ***Paracoccus*** spp**. Tmm, trimethylamine monooxygenase; Dmm, putative dimethylamine monooxygenase; NMGP, *N*-methylglutamate pathway; Mau, methylamine dehydrogenase; Mxa, Mxa-type methanol dehydrogenase; Xox, Xox-type methanol dehydrogenase; GSH, glutathione; CH_2_ = THF, 5,10-methylene-tetrahydrofolate; FolD, 5,10-methylene-tetrahydrofolate dehydrogenase/5,10-methylene-tetrahydrofolate cyclohydrolase; PurU, formyltetrahydrofolate deformylase; FtfL, formate-tetrahydrofolate ligase. The accession numbers of *Paracoccus* complete genomes: JCM7686 – GQ410978, GQ468939, GQ468938, CP006650–CP006655, and Pd1222 – CP000489–CP000491. The accession numbers of *Paracoccus* draft genomes: J39 – JAEN01000001–JAEN01000050, J55 – AZVA01000001–AZVA01000069, N5 – AQUO01000001–AQUO01000003, ATCC BAA-599 – JHWH01000001– JHWH01000073, DSM 582 – JRKO01000001– JRKO01000187, TRP – AEPN01000001– AEPN01000119, J40 – JAGK01000001– JAGK01000119, J46 – JAEM01000001– JAEM01000105, HAMBI 3106 – JRKS01000001– JRKS01000137, 10990 – JRKR01000001– JRKR01000309, 39524 – JRKP01000001– JRKP01000273, 4681 – JRKT01000001– JRKT01000176, 5503 – JRKQ01000001– JRKQ01000265, JCM 14014 – JRKN01000001– JRKN01000122, and ATCC 21588 – ATUJ01000001– ATUJ01000035.

Annotated genomic sequences of 17 strains of *Paracoccus* spp. (including *P. aminophilus* JCM 7686) are currently available in the GenBank database (Figure 6). The genomes of these strains were screened for the presence of 67 genes encoding enzymes involved in methylotrophy that have been identified in *P. aminophilus* and other methylotrophic *Alphaproteobacteria*. It is important to note that for 15 *Paracoccus* spp. strains only draft genomes were available, thus some data may be missing. Therefore, the lack of the particular genes has to be verified after obtaining complete genome sequences of those strains.

On the basis of the performed analysis, strains of *Paracoccus* spp. were classified into four groups: (i) autotrophic methylotrophs, which can potentially assimilate CO_2_ via the Calvin cycle (six strains), (ii) facultatively autotrophic methylotrophs, which have both the serine cycle and the Calvin cycle (3 strains), (iii) heterotrophic methylotrophs, which use only the serine pathway for carbon assimilation, represented only by *P. aminophilus* JCM 7686, and (iv) non-methylotrophs (Figure 6).

More differences were observed when the strains were compared in terms of the range of C1 compounds that potentially can be oxidized. All but one strain (*P. halophilus* JCM 14014) encode XoxF methanol dehydrogenase, and six strains (*P. denitrificans* Pd1222, *P. pantotrophus* J40, J46 and *Paracoccus* spp. J39, J55, TRP) also encode a PQQ-dependent calcium-binding methanol dehydrogenase (MxaFI) (Figure 6). Eight of the analyzed strains encode enzymes responsible for the utilization of methylated amines. Of these, five (JCM 7686, J39, J55, N5, and ATCC BAA-599) also encode enzymes that are required for trimethylamine oxidation via trimethylamine *N*-oxide. In addition, these five strains plus *P. versutus* DSM 582 possess *dmmABCD* genes encoding putative dimethylamine monooxygenase (Figure 6).

Another interesting observation was made while analyzing genes involved in methylamine utilization. Three groups of strains were distinguished that are likely to be able to perform methylamine oxidation via different routes: (i) the NMGP (all facultatively autotrophic methylotrophs, i.e., *Paracoccus* sp. J39, J55, and N5), (ii) the pathway involving Mau (*P. denitrificans* Pd1222 and *Paracoccus* sp. TRP), and (iii) both pathways (*P. aminophilus, P. yeei*, and *P. versutus*) (Figure 6).

**Figure 7:**
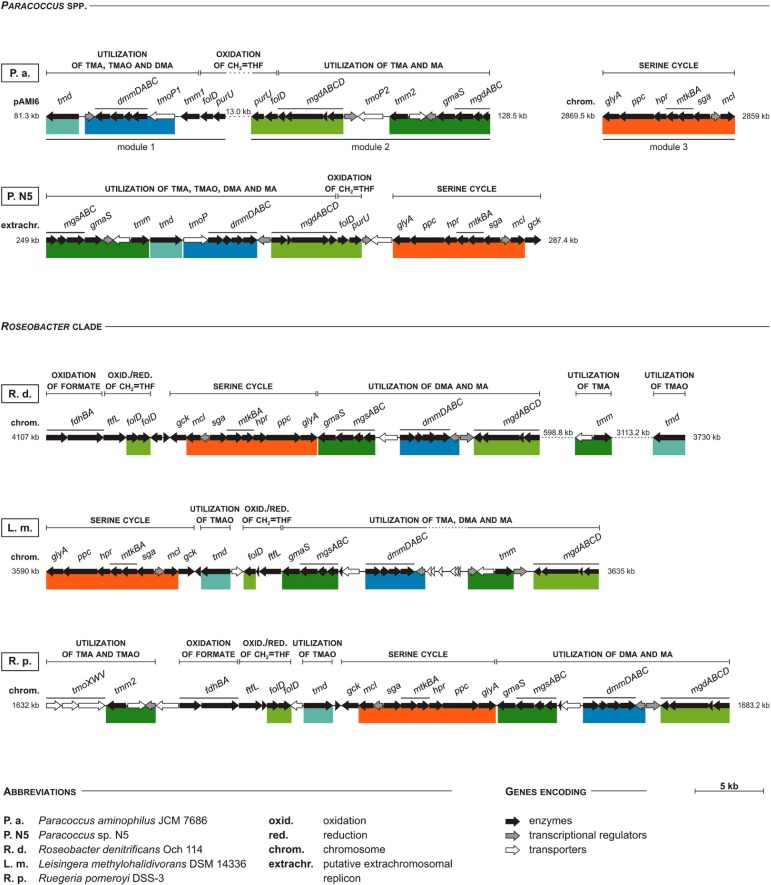
**Comparison of clustering of the serine pathway genes with other methylotrophy-linked genes in ***P. aminophilus*** JCM 7686, ***Paracoccus*** sp. N5, and three selected strains of the ***Roseobacter*** clade**. Colored bars show shuffling of specific DNA regions when compared to *P. aminophilus* JCM 7686. In *P. aminophilus* JCM 7686, the presented set of methylotrophy genes is divided into three clusters, two located within pAMI6 and one within the chromosome. In the other strains all these genes constitute a single methylotrophy island, which may be located within the chromosome (*R. pomeroyi* DSS-3 and *L. methylohalidivorans* DSM 14336), or in putative extrachromosomal replicons (*Paracoccus* sp. N5). In some cases, certain genes of the methylotrophy island may be found in a distant location in a genome (e.g., the *tmm* and *tdm* genes of *R. denitrificans* Och 114). The full names of enzymes encoded by most of the genes presented in the figure are summarized in Table S4. The others are *tmoP*—TMAO permease gene (Zhu et al., 2014), and *tmoXWV*—TMAO ABC-transporter system genes (Lidbury et al., 2014). The accession numbers of particular replicons are as follows: JCM 7686 – CP006654 (pAMI6) and CP006650 (chromosome), N5 – AQUO01000003, Och 114 – CP000362, DSM 14336 – CP006773, DSS-3 – CP000031.

What is noteworthy, in all strains possessing the serine cycle genes, a co-occurrence of the genes involved in TMA oxidation via TMAO and the NMGP was observed. In *P. aminophilus* JCM 7686, these genes are present within three separate clusters. Two of them are located in pAMI6 (genes involved in TMA oxidation and in the NMGP) and the other (involved in the serine cycle) in the chromosome (Figure 7). In *Paracoccus* sp. N5 all the aforementioned genes form a single methylotrophy island, which is most probably extrachromosomally located (Figure 7).

Interestingly, we found that the gene clusters homologous with the methylotrophy island of *Paracoccus* sp. N5 are also present in the chromosomes of numerous strains of the marine *Roseobacter* clade (Figure 7), many of which were recognized as serine cycle methylotrophs (Newton et al., 2010). These bacteria comprise up to 20% of the microorganisms in coastal surface waters (Chen, 2012). Therefore, the identified methylotrophy islands may constitute one of the most abundant sets of genes participating in C1 metabolism worldwide. Since these islands contain genes involved in all three stages of the methylotrophy process (oxidation of specific C1 substrates, oxidation of CH_2_ = THF and assimilation of C1 units), their transfer to other hosts may result in the conversion of non-methylotrophic strains into methylotrophs. It is noteworthy that bacteria of the *Roseobacter* clade are well known for the presence of numerous conjugative megaplasmids, which may promote horizontal transmission of large segments of genomic DNA (Petersen et al., 2012, 2013).

In the case of *P. aminophilus* JCM 7686 several other extrachromosomal elements besides pAMI6 (plasmids pAMI1, pAMI2, pAMI4, and chromid pAMI5) contain genes linked to methylotrophy. As previously shown (Dziewit et al., 2014), the host range of these elements is not limited to *Paracoccus* spp. and extends to other strains of *Alphaproteobacteria*, including *Agrobacterium tumefaciens* and *Rhizobium etli*. Thus, further transfer of these extrachromosomally-located methylotrophy genes to other hosts may result in the formation of “patchwork” methylotrophic pathways and the generation of bacterial strains with novel metabolic properties.

## Conclusions

The major goal of this study was to define the metabolic network involved in the C1 metabolism of *P. aminophilus* JCM 7686 and to compare it at the genetic level with other members of the genus *Paracoccus*. A genome-wide analysis revealed the great methylotrophic potential of this strain, manifested in its ability to utilize a wide range of C1 compounds, including formamide and *N*,*N*-dimethylformamide. These phenotypes enabled adaptation of *P. aminophilus* to its natural “methylotrophic” habitat, which was soil contaminated with DMF.

On the basis of the *in silico* and experimental evidence we defined complex methylotrophic pathways of *P. aminophilus*, with by-pass pathways for methylamine utilization and genes encoding multiple enzymes of the same specificity involved e.g., in trimethylamine utilization. The analysis revealed that this strain is the only known heterotrophic methylotroph among *Paracoccus* spp., encoding enzymes of the serine cycle as an exclusive pathway for C1 unit assimilation. Thus, *P. aminophilus* JCM 7686 is an excellent model for the studies on genetic diversity and evolution of methylotrophy in this group of bacteria.

### Conflict of interest statement

The authors declare that the research was conducted in the absence of any commercial or financial relationships that could be construed as a potential conflict of interest.

## Supplementary material

Figure S1 **Chemical structures of C1 compounds utilized by *P. aminophilus* JCM 7686**.

Figure S2 **Effect of the *mauA* mutation on growth of *P. aminophilus* JCM 7686 on methylamine**. wt, wild type; *mauA*, *mauA* insertional mutant. The values are means of three replicates, and the error bars indicate the standard deviations.

Table S1 **Bacterial strains used in this study**.

Table S2 **Plasmids used and constructed in this study**.

Table S3 **Oligonucleotide primers used in this study**.

Table S4 **Genes of *P. aminophilus* JMC 7686 involved in the metabolism of C1 compounds**.
