# Supplementary figures and images for: Genome-guided insight into the methylotrophy of Paracoccus aminophilus JCM 7686
Source: Front Microbiol. 2015 Aug 21;6:852. doi: 10.3389/fmicb.2015.00852 (PMC4543880; doi:10.3389/fmicb.2015.00852)

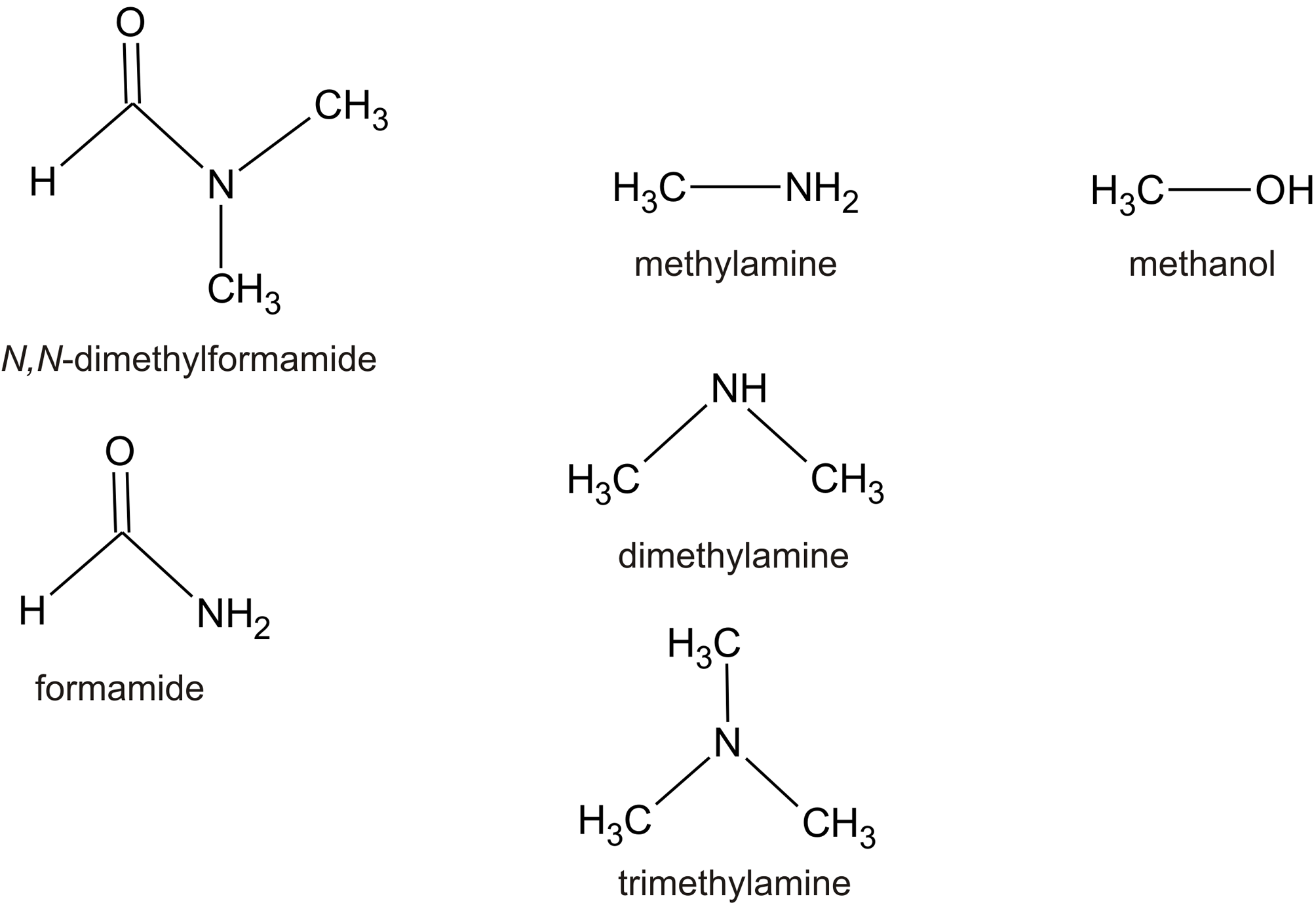

Supplement: Figure S1 — Chemical structures of C1 compounds utilized by P. aminophilus JCM 7686. [file Image1.TIF]

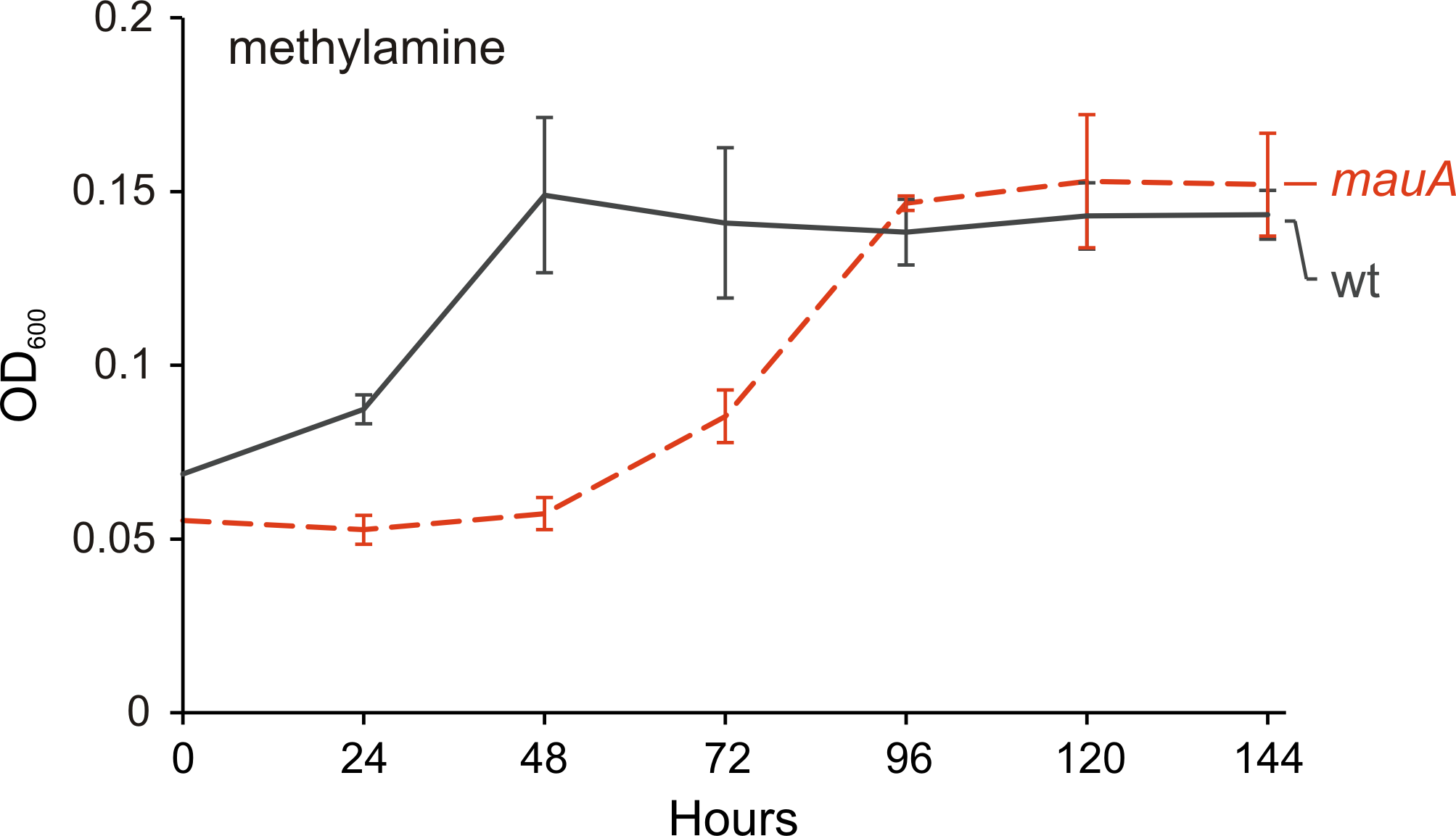

Supplement: Figure S2 — Effect of the mauA mutation on growth of P. aminophilus JCM 7686 on methylamine. wt, wild type; mauA, mauA insertional mutant. The values are means of three replicates, and the error bars indicate the standard deviations. [file Image2.TIF]
