# Supplementary material for: Genome-guided insight into the methylotrophy of Paracoccus aminophilus JCM 7686
Source: Front Microbiol. 2015 Aug 21;6:852. doi: 10.3389/fmicb.2015.00852 (PMC4543880; doi:10.3389/fmicb.2015.00852)
Supplement: Table S1 — Bacterial strains used in this study. [file Table1.DOCX]

**Table S1.** Bacterial strains used in this study.

| **Strain** | **Characteristics** | **References** |
| --- | --- | --- |
| *Escherichia coli* TG1 | F' [*traD36 proAB^+^ lacI^q^ lacZΔM15*] *supE44 thi-1 Δ(lac-proAB) Δ(mcrB-hsdSM)5*; strain used for blue/white cloning and as a donor of plasmids in triparental mating | (Gibson, 1984) |
| *E. coli* S17-1 | F^-^, RP4-2(Km::Tn*7*,Tc::Mu-1), *pro*-82, *recA*1, *endA*1, *thiE*1, *hsdR*17, *creC*510, λ*pir* lysogen; strain used for maintaining pDS132 and its derivatives and as a donor of plasmids in biparental mating | (Simon et al., 1983) |
| *Paracoccus aminophilus* JCM 7686R | Rif^r^ derivative of wild-type strain JCM 7686 | (Bartosik et al., 2002) |
| *P. aminophilus* *xoxF* | JCM 7686R derivative, *xoxF* (JCM7685_0090)::Km | This study |
| *P. aminophilus xoxF* compl. | JCM 7686R derivative, *xoxF* (JCM7685_0090)::Km, pBBRKm-*xoxF* | This study |
| *P. aminophilus* *dmmA* | JCM 7686R derivative, *dmmA* (JCM7685_ pAMI6p073)::Km | This study |
| *P. aminophilus* *dmmB* | JCM 7686R derivative, *dmmB* (JCM7685_ pAMI6p072)::Km | This study |
| *P. aminophilus* *dmmC* | JCM 7686R derivative, *dmmC* (JCM7685_ pAMI6p071)::Km | This study |
| *P. aminophilus* *dmmD* | JCM 7686R derivative, *dmmD* (JCM7685_ pAMI6p074)::Km | This study |
| *P. aminophilus mauA* | JCM 7686R derivative, *mauA* (JCM7685_ JCM7686_0163)::Km | This study |
| *P. aminophilus tmm1* | JCM 7686R derivative, *tmm1* (JCM7685_ pAMI6p076)::Km | This study |
| *P. aminophilus tmm2* | JCM 7686R derivative, *tmm2* (JCM7685_ pAMI6p102)::Km | This study |
| *P. aminophilus tmm12* | JCM 7686R derivative, *tmm1* (JCM7685_ pAMI6p076)::Tc, *tmm2* (JCM7685_ pAMI6p102)::Km | This study |

References:

Bartosik, D., Baj, J., Bartosik, A.A., and Wlodarczyk, M. (2002). Characterization of the replicator region of megaplasmid pTAV3 of *Paracoccus versutus* and search for plasmid-encoded traits. Microbiology 148, 871-881.

Gibson, T.J. (1984). Studies on Epstein-Barr genome. PhD thesis, University of Cambridge.

Simon, R., Priefer, U., and Pühler, A. (1983). A broad host range mobilization system for invivo genetic-engineering - transposon mutagenesis in gram-negative bacteria. Bio-Technology 1, 784-791.
