# Supplementary material for: Genome-guided insight into the methylotrophy of Paracoccus aminophilus JCM 7686
Source: Front Microbiol. 2015 Aug 21;6:852. doi: 10.3389/fmicb.2015.00852 (PMC4543880; doi:10.3389/fmicb.2015.00852)
Supplement: Table S2 — Plasmids used and constructed in this study. [file Table2.DOCX]

**Table S2.** Plasmids used in this study.

| **Plasmid** | **Characteristics/construction details** | **Applications** | **Reference** |
| --- | --- | --- | --- |
| pBBR1MCS-3 | Tc^r^, *ori* pBBR1 (broad host range, replication in both *Gamma-* and *Alphaproteobacteria*), *oriT* RK2 (mobilizable), *lacZ*α, MCS | blue/white cloning, complemention of mutations, delivered into *P. aminophilus* JCM 7686 cells via triparental mating | Kovacs  et al., 1994 |
| pBBR1MCS-5 | Gm^r^, *ori* pBBR1 (broad host range, replication in both *Gamma-* and *Alphaproteobacteria*), *oriT* RK2 (mobilizable), *lacZ*α, MCS | blue/white cloning, complemention of mutations, delivered into P. aminophilus JCM 7686 cells via triparental mating | Kovacs  et al., 1994 |
| pDIY-KM | Km^r^, Ap^r^, *ori* ColE1 | source of kanamycin resistance gene in construction of cassettes for mutagenesis | Dziewit  et al., 2011 |
| pDS132 | Cm^r^; *ori* R6K (narrow host range, replication only in *E. coli* λpir);  *oriT* RK2 (mobilizable); *sacB* (a counter-selection gene enabling selection of double-cross mutants on appropriate medium with sucrose) | delivery of cassettes for mutagenesis into  *P. aminophilus* JCM 7686R cells via biparental mating | Philippe  et al., 2004 |
| pKRP12 | Tc^r^, Ap^r^, *ori* ColE1 | source of tetracycline resistance gene in construction of cassettes for mutagenesis | Reece and Phillips, 1995 |
| pRK2013 | Km^r^, the conjugal transfer system of RK2 | helper plasmid used to mobilize mobilizable vectors in triparental mating | Ditta et al.  1980 |
| pF4.1.D | pCC1FOS (Epicentre) derivative carrying 36-kb fragment of pAMI6 (fragment between 72,976 and 109,240 bp); the phosmid selected from phosmid genomic library of *P. aminophilus* JCM 7686 using PCR with LLPHMOT1 and LRPHMOT1 primers (Table S3) | source of *dmmDABC* operon for cloning | This study |
| pBBR-*dmmDABC* | pBBR1MCS-3 derivative carrying 4.8-kb restriction fragment of pF4.1.D (cut with StuI) including *dmmDABC*, the fragment was cloned into SmaI site of pBBR1MCS-3 | source of *dmmABC* genes in construction of cassettes for mutagenesis | This study |
| pBBR-*tmm1* | pBBR1MCS-5 derivative carrying 1.6-kb PCR product obtained with LMOT1XB and RMOT1KP primers (Table S3) cloned between KpnI and XbaI sites of pBBR1MCS-5 | complementation of *tmm1* mutation | This study |
| pBBR-*tmm2* | pBBR1MCS-5 derivative carrying 1.6-kb PCR product obtained with LMOT2XB and RMOT2KP primers (Table S3) cloned between KpnI and XbaI sites of pBBR1MCS-5 | complementation of *tmm2* mutation | This study |
| pBBR-*xoxF* | pBBR1MCS-3 derivative carrying wild type *xoxF* gene - 2.0-kb PCR product obtained with LxoxKpn and RxoxXba primers (Table S3) cloned between KpnI and XbaI sites of pBBR1MCS-3) | complementation of *xoxF* mutation | This study |
| pDS-*dmmA*::Km | pDS132 derivative carrying 2.6-kb cassette for *dmmA* mutagenesis (Km^r^); the cassette was constructed by cloning 1.9-kb restriction fragment of pBBR-*dmmDABC* (cut with PstI and SacI) containing *dmmA* gene into pDS132 (between PstI and SacI sites), resultant plasmid was then cut with NdeI and PvuI (creating 280-bp deletion in *dmmA* gene), 3’-sticky ends were blunted with T4 DNA polymerase and kanamycin resistance gene, cut from pDYI-KM with SmaI, was cloned into truncated *dmmA* gene | mutagenesis of *dmmA* gene | This study |
| pDS-*dmmB*::Km | pDS132 derivative carrying 4.0-kb cassette for *dmmB* mutagenesis (Km^r^); the cassette was constructed by cloning 3.1-kb restriction fragment of pBBR-*dmmDABC* (cut with PstI) containing *dmmB* gene into pDS132 (into PstI site), resultant plasmid was then cut with ApaLI and RsrII (creating 400-bp deletion in *dmmB* gene), 5’-sticky ends were blunted with DNA polymerase I Klenow fragment and kanamycin resistance gene, cut from pDIY-KM with SmaI, was cloned into truncated *dmmB* gene | mutagenesis of *dmmB* gene | This study |
| pDS-*dmmC*::Km | pDS132 derivative carrying 2.0-kb DNA cassette for *dmmC* mutagenesis (Km^r^); the cassette was constructed by cloning 1.7-kb restriction fragment of pBBR-*dmmDABC* (cut with PstI and SacI) into pDS132 (between PstI and SacI sites), resultant plasmid was then cut with PvuI and NdeI (creating 600-bp deletion in *dmmC* gene), 3’-sticky ends were blunted with T4 DNA polymerase and kanamycin resistance gene, cut from pDIY-KM with SmaI, was cloned into truncated *dmmC* gene | mutagenesis of *dmmC* gene | This study |
| pDS-*dmmD*::Km | pDS132 derivative carrying 1.8-kb cassette for *dmmD* mutagenesis (Km^r^) constructed by overlap extension PCR with appropriate primers and cloned between two XbaI sites of the vector | mutagenesis of *dmmD* gene | This study |
| pDS-*mauA*::Km | pDS132 derivative carrying 1.7-kb cassette for *mauA* mutagenesis (Km^r^) constructed by overlap extension PCR with appropriate primers (Table S3) and cloned between XbaI and SacI sites of the vector | mutagenesis od *mauA* gene | This study |
| pDS-*tmm1*::Km | pDS132 derivative carrying 1.8-kb cassette for *tmm1* mutagenesis (Km^r^) constructed by overlap extension PCR with appropriate primers (Table S3) and cloned between XbaI and SacI sites of the vector | mutagenesis of *tmm1* gene | This study |
| pDS-*tmm1*::Tc | pDS132 derivative carrying 2.5-kb cassette for *tmm1* mutagenesis (Tc^r^) constructed by overlap extension PCR with appropriate primers (Table S3) and cloned between XbaI and SacI sites of the vector | mutagenesis of *tmm1* gene | This study |
| pDS-*tmm2*::Km | pDS132 derivative carrying 1.8-kb cassette for *tmm2* mutagenesis (Km^r^) constructed by overlap extension PCR with appropriate primers (Table S3) and cloned between XbaI and SacI sites of the vector | mutagenesis of *tmm2* gene | This study |
| pDS-*xoxF*::Km | pDS132 derivative carrying 2.4-kb cassette for *xoxF* mutagenesis (Km^r^); the cassette was constructed by cloning 2.1-kb SacI restriction fragment of PCR product obtainted with 1xox and 6xox primers (Table S3) into pDS132 (into SacI site), resultant plasmid was then cut with PvuI (creating 620-bp deletion in *xoxF* gene), 3’-sticky ends were blunted with T4 DNA polymerase and kanamycin resistance gene, cut from pDYI-KM with SmaI, was cloned into truncated *xoxF* gene | mutagenesis of *xoxF* gene | This study |
