# Supplementary material for: Genome-guided insight into the methylotrophy of Paracoccus aminophilus JCM 7686
Source: Front Microbiol. 2015 Aug 21;6:852. doi: 10.3389/fmicb.2015.00852 (PMC4543880; doi:10.3389/fmicb.2015.00852)
Supplement: Table S3 — Oligonucleotide primers used in this study. [file Table3.DOCX]

**Table S3.** Oligonucleotide primers used in this study.

| **Name** | **Sequence** | **Application** |
| --- | --- | --- |
| 1xox | ATGAGCTCAAATCGGTCTCGGCCTTCGC | construction of cassette for mutagenesis of *xoxF* |
| 6xox | ATGAGCTCCGGCCTTGCTGACATCTTGC |  |
| 0xox | CGTGGAGCCCGCATTTCAAG | confirmation of correct introduction of *xoxF* mutation |
| 7xox | GGGCGAGCAGATAAGCGTAG |  |
| LxoxKpn | ATGGTACCCCGGATGCAGGAAGGTTACG | amplification of wild type *xoxF* gene for complementation |
| RxoxXba | GCTCTAGAGGCCTAACTGCCTCTTAGCC |  |
| LLPHMOT1 | CGTCTAGAATGACCAAACGAGTGG | construction of cassette for mutagenesis of *tmm1* (Km^r^) using overlap extension PCR |
| LRPHMOT1 | TGAGACACAACGTGGCATCATAGG |  |
| KLPHMOT1 | CCTATGATGCCACGTTGTGTCTCA |  |
| KRPHMOT1 | ACTGATCCTGCGCCAGTGTTACAA |  |
| RLPHMOT1 | TTGTAACACTGGCGCAGGATCAGT |  |
| RRPHMOT1 | TAGAGCTCAGTTGCGCAGGTAG |  |
| LRMOT1TC | GACACTATAGAACCATGCGGATCG | construction of cassette for mutagenesis of *tmm1* (Tc^r^) using overlap extension PCR (LLPHMOT1 and RRPHMOT1 were also used for this purpose) |
| TLPHMOT1 | CGATCCGCATGGTTCTATAGTGTC |  |
| TRPHMOT1 | TGAACCACTGATGTTGGTTTGCGC |  |
| RLMOT1TC | GCGCAAACCAACATCAGTGGTTCA |  |
| LMOT1XB | TAGGTACCGCGTGACGGCAAACAGGTTC | confirmation of correct introduction of *tmm1* mutations and amplification of wild type *tmm1* gene for complementation |
| RMOT1KP | GATCTAGAGGATCAGTTGCGCAGGTAGC |  |
| LLPHMOT2 | CGTCTAGAATGACGAAGCGAGTCGA | construction of cassette for mutagenesis of *tmm2* |
| LRPHMOT2 | TGAGACACAACGGCGGATCGAACT |  |
| KLPHMOT2 | AGTTCGATCCGCCGTTGTGTCTCA |  |
| KRPHMOT2 | CACTGATCCTGCGCCAGTGTTACA |  |
| RLPHMOT2 | TGTAACACTGGCGCAGGATCAGTG |  |
| RRPHMOT2 | GCGAGCTCCAGTTCTTCAGATAGG |  |
| LMOT2KP | TAGGTACCCAGATTTCGCGGCAGGGTTC | confirmation of correct introduction of *tmm2* mutation and amplification of wild type *tmm2* gene for complementation |
| RMOT2XB | GATCTAGACCGCAGAGTCACGTCAGTTC |  |
| 1dmmami6 | GATCTAGACTTTGGACGACGCCGATCAG | construction of cassette for mutagenesis of *dmmD* |
| 2dmmami6 | TTTGAGACACAACGTCCGCAGCGCCAAG |  |
| 3dmmami6 | CTTGGCGCTGCGGACGTTGTGTCTCAAA |  |
| 4dmmami6 | GCTCGAGGACTGGAGCCAGTGTTACAAC |  |
| 5dmmami6 | GTTGTAACACTGGCTCCAGTCCTCGAGC |  |
| 6dmmami6 | AGTCTAGAGCCTATCGCCATTGGGTGAC |  |
| 0dmmami6 | CGGTGATGCCTTTGCAATGG | confirmation of correct introduction of *dmmD* mutation |
| 7dmmami6 | TTTCGTCAATGCGCTGTTCC |  |
| LLPHMAUA | CGTCTAGAACCGAATCAGGCCTGTT | construction of cassette for mutagenesis of *mauA* using overlap extension PCR |
| LRPHMAUA | TGAGACACAACGAAAGCCAGCTGA |  |
| KLPHMAUA | TCAGCTGGCTTTCGTTGTGTCTCA |  |
| KRPHMAUA | GGAGGATAATCGGCCAGTGTTACA |  |
| RLPHMAUA | GTAACACTGGCCGATTATCCTCCC |  |
| RRPHMAUA | TCGAGCTCGATCATCAAGTCGATC |  |
| LMAUA | ATCGACGCAGGGTCGCTTTC | confirmation of correct introduction of *mauA* mutation |
| RMAUA | TCGAGCTGCCCGATTACTTC |  |
| Tmm13L | GTGCCTGTGCCGCTTTCTTC | RT-qPCR for transcript of *tmm1* gene |
| Tmm13R | GTCTTCCAGCGCTTCGTTCC |  |
| Tmm23L | AGGGCGTCGTCTACGTCAAC | RT-qPCR for transcript of *tmm2* gene |
| Tmm23R | TCCGGCCCAGGATAATGTCG |  |
| AceA1L | CGAAACCGACACGCCGAATG | RT-qPCR for transcript of *aceA* gene |
| AceA1R | GCACCTGTTTGCGCAGGTTG |  |
| Hpr2L | CGGCGAGCCGAACATCAATC | RT-qPCR for transcript of *hpr* gene |
| Hpr2R | TCACGGCCGGCAAAGAAGTC |  |
| Gck3L | CGCGCTGCTGATTTCCGATG | RT-qPCR for transcript of *gck* gene |
| Gck3R | CGTCGAGATGCCAGCGTTTG |  |
| Ecm3L | CGCACCTATGCGGGTCATTC | RT-qPCR for transcript of *ecm* gene |
| Ecm3R | GTCATAGCCGGTCTGGGTTG |  |
| Mcm2L | ACGCCTTCTATCGCCGCAAC | RT-qPCR for transcript of *mcm* gene |
| Mcm2R | TGCCGACATCGCCTTCAACG |  |
| RpoA3L | TCAAGGGCGTGACGCTGAAG | RT-qPCR for transcript of *rpoA* gene (reference) |
| RpoA3R | GTGATCGCGGTTCAGGATGG |  |
