# Supplementary material for: Genome-guided insight into the methylotrophy of Paracoccus aminophilus JCM 7686
Source: Front Microbiol. 2015 Aug 21;6:852. doi: 10.3389/fmicb.2015.00852 (PMC4543880; doi:10.3389/fmicb.2015.00852)
Supplement: Table S4 — Genes of P. aminophilus JMC 7686 involved in the metabolism of C1 compounds. [file Table4.DOCX]

**Table S4.** Genes of *Paracoccus aminophilus* JMC 7686 involved in the metabolism of C1 compounds.

| Gene(s) | Replicon(s) | Encoded enzyme | EC number |
| --- | --- | --- | --- |
| Oxidation of C1 substrates | | | |
| JCM7686_pAMI6p076  JCM7686_pAMI6p102 | pAMI6 | trimethylamine monooxygenase (Tmm) | 1.14.13.8 |
| JCM7686_pAMI6p069 | pAMI6 | trimethylamine-*N-*oxide demethylase (Tdm) | 4.1.2.32 |
| JCM7686_pAMI2p015-016 | pAMI2 | *N,N*-dimethylformamidase (DmfA1A2) | 3.5.1.56 |
| JCM7686_pAMI6p071-074 | pAMI6 | putative dimethylamine monooxygenase (DmmABCD)^1^ | - |
| JCM7686_0162-0171 | chromosome | methylamine dehydrogenase (MauFAEDBCJGMN) | 1.4.9.1 |
| JCM7686_pAMI6p105 | pAMI6 | glutamate-methylamine ligase (GmaS) | 6.3.4.12 |
| JCM7686_pAMI6p106-108 | pAMI6 | *N*-methyl-L-glutamate synthase (MgsABC) | 2.1.1.21 |
| JCM7686_pAMI6p096-099 | pAMI6 | *N*-methyl-L-glutamate dehydrogenase  (MgdABCD) | 1.5.99.5 |
| JCM7686_0090 | chromosome | methanol dehydrogenase (XoxF) | 1.1.2.7 |
| JCM7686_0085 | chromosome | *S*-(hydroxymethyl)glutathione synthase (Gfa) | 4.4.1.22 |
| JCM7686_0086 | chromosome | *S*-(hydroxymethyl)glutathione dehydrogenase (FlhA) | 1.1.1.284 |
| JCM7686_0089 | chromosome | *S*-formylglutathione hydrolase (FghA) | 3.1.2.12 |
| JCM7686_pAMI6p077  JCM7686_pAMI6p095 JCM7686_pAMI5p256 | pAMI6, pAMI5 | 5,10-methylene-tetrahydrofolate dehydrogenase/ 5,10-methylene-tetrahydrofolate cyclohydrolase (FolD) | 1.5.1.5/  3.5.4.9 |
| JCM7686_pAMI6p078  JCM7686_pAMI6p094 JCM7686_pAMI5p257 | pAMI6, pAMI5 | formyltetrahydrofolate deformylase (PurU) | 3.5.1.10 |
| Oxidation of C1 substrates | | | |
| JCM7686_pAMI6p042 | pAMI6 | formate-tetrahydrofolate ligase (FtfL) | 6.3.4.3 |
| JCM7686_pAMI4p036  JCM7686_1450 | chromosome, pAMI4 | formamidase (FmdA) | 3.5.1.49 |
| JCM7686_0639-0643  JCM7686_3476-3480  JCM7686_2088  JCM7686_pAMI1p027 | chromosome, pAMI1 | formate dehydrogenase (Fdh) | 1.2.1.2 |
| Serine cycle | | | |
| JCM7686_2770  JCM7686_2900 | chromosome | glycine hydroxymethyltransferase (GlyA) | 2.1.2.1 |
| JCM7686_1647  JCM7686_2765 | chromosome | serine-glyoxylate transaminase (Sga) | 2.6.1.45 |
| JCM7686_2768 | chromosome | hydroxypyruvate reductase (Hpr) | 1.1.1.29 |
| JCM7686_pAMI5p028 | pAMI5 | glycerate 2-kinase (Gck) | 2.7.1.165 |
| JCM7686_1636 | chromosome | enolase (Eno) | 4.2.1.11 |
| JCM7686_2769 | chromosome | phosphoenolpyruvate carboxylase (Ppc) | 4.1.1.31 |
| JCM7686_2576 | chromosome | malate dehydrogenase (Mdh) | 1.1.1.37 |
| JCM7686_2766-2767 | chromosome | malate-CoA ligase (MtkAB) | 6.2.1.9 |
| JCM7686_2763  JCM7686_3168 | chromosome | malyl-CoA lyase (Mcl)^2^ | 4.1.3.24 |
| Ethylmalonyl-CoA pathway | | | |
| JCM7686_0515 | chromosome | acetyl-CoA C-acetyltransferase (PhaA) | 2.3.1.9 |
| Ethylmalonyl-CoA pathway | | | |
| JCM7686_0514 | chromosome | acetoacetyl-CoA reductase (PhaB) | 1.1.1.36 |
| JCM7686_1380 | chromosome | (*R*)-3-hydroxybutyryl-CoA dehydratase (CroR) | 4.2.1.55 |
| JCM7686_1036 | chromosome | crotonyl-CoA carboxylase/reductase (Ccr) | 1.3.1.85 |
| JCM7686_2438 | chromosome | ethylmalonyl-CoA epimerase (Epi)^3^ | 5.1.99.1 |
| JCM7686_1037 | chromosome | ethylmalonyl-CoA mutase (Ecm) | - |
| JCM7686_0656 | chromosome | (2*S*)-methylsuccinyl-CoA dehydrogenase (Msd) | - |
| JCM7686_2571 | chromosome | mesaconyl-CoA hydratase (Mcd) | 4.2.1.148 |
| JCM7686_2763  JCM7686_3168 | chromosome | β-methylmalyl-CoA lyase (Mcl)^2^ | 4.1.3.24 |
| JCM7686_1909  JCM7686_1905 | chromosome | propionyl-CoA carboxylase (PccAB) | 6.4.1.3 |
| JCM7686_2438 | chromosome | methylmalonyl-CoA epimerase (Epi)^3^ | 5.1.99.1 |
| JCM7686_1912 | chromosome | methylmalonyl-CoA mutase (Mcm) | 5.4.99.2 |
| Glyoxylate shunt | | | |
| JCM7686_pAMI6p121 | pAMI6 | isocitrate lyase (AceA) | 4.1.3.1 |
| JCM7686_pAMI6p120 | pAMI6 | malate synthase (AceB)^4^ | 2.3.3.9 |
| JCM7686_1627 | chromosome | malate synthase G (GlcB)^4^ | 2.3.3.9 |

^1^ Dimethylamine monooxygase activity of the purified products of the genes was not demonstrated. The role of the genes and their close homologs from *Methylocella silvestris* in dimethylamine metabolism was confirmed only by growth analyses of knockout mutants conducted in this study and by Zhu and co-workers (Zhu et al., 2014).

^2^ Mcl is an enzyme catalyzing both the cleavage of malyl-CoA into glyoxylate and acetyl-CoA and the cleavage of β-methylmalyl-CoA into glyoxylate and propionyl-CoA (Erb et al., 2010).

^3^ Epi is an enzyme catalyzing the conversion of both (2S)-ethylmalonyl-CoA and (2S)-methylmalonyl-CoA into their 2R-stereoisomers (Erb et al., 2008).

^4^ The genome of *Paracoccus aminophilus* JCM 7686 encodes two putative non-homologous malate synthases.
